# The exon junction complex senses energetic stress and regulates contractility and cell architecture in cardiac myocytes

**DOI:** 10.1042/BSR20170707

**Published:** 2017-07-07

**Authors:** Olivier A. Pierrat, Anju Paudyal, James Woodruff, Olga Koroleva, Samuel Y. Boateng

**Affiliations:** School of Biological Sciences, University of Reading, Whiteknights, Reading, Berkshire, U.K.

**Keywords:** cardiomyocytes, Exon junction complex, hypoxia, metabolic stress, myocardium, myocardial infarction

## Abstract

The exon junction complex (EJC) is the main mechanism by which cells select specific mRNAs for translation into protein. We hypothesized that the EJC is involved in the regulation of gene expression during the stress response in cardiac myocytes, with implications for the failing heart. In cultured rat neonatal myocytes, we examined the cellular distribution of two EJC components eukaryotic translation initiation factor 4A isoform 3 (eIF4A3) and mago nashi homologue (Mago) in response to metabolic stress. There was significant relocalization of eIF4A3 and Mago from the nucleus to cytoplasm following 18 h of hypoxia. Treating myocytes with 50 mM NaN_3_ for 4 h to mimic the metabolic stress induced by hypoxia also resulted in significant relocalization of eIF4A3 and Mago to the cytoplasm. To examine whether the effects of metabolic stress on the EJC proteins were dependent on the metabolic sensor AMP kinase (AMPK), we treated myocytes with 1 μM dorsomorphin (DM) in combination with NaN_3_. DM augmented the translocation of Mago and eIF4A3 from the nucleus to the cytoplasm. Knockdown of eIF4A3 resulted in cessation of cell contractility 96 h post-treatment and a significant reduction in the number of intact sarcomeres. Cell area was significantly reduced by both hypoxia and eIF4A3 knockdown, whilst eIF4A3 knockdown also significantly reduced nuclear size. The reduction in nuclear size is unlikely to be related to apoptosis as it was reversed in combination with hypoxia. These data suggest for the first time that eIF4A3 and potentially other EJC members play an important role in the myocyte stress response, cell contractility and morphology.

## Introduction

The adult heart responds to stress by undergoing biochemical and molecular changes which result in adaptation. Stresses such as haemodynamic overload or hypoxia trigger a response which leads to the expression of genes associated with early stages of heart development, called the foetal gene pattern [1]. The foetal gene pattern is beneficial in the short term because it increases the efficiency of the heart and enables it to cope with the stress [2]. However, in the long term, the foetal pattern of gene expression leads to maladaptive remodelling and heart failure by a mechanism which is still poorly understood.

Others have examined the regulation of gene expression in the heart, and in many cases found no correlation between mRNA and protein levels [3]. Protein expression does not only depend on transcriptional activity in the failing heart, but also occurs at the post-translational level. Several variants of mRNA can be made from the same gene and several genes may code isoforms of similar proteins, and regulated processes select which of these mRNAs are processed for translation into protein [4]. The post-transcriptional regulation of gene expression becomes particularly important for the selection of spliced isoforms needed in different physiological conditions, for cellular adaptation to environmental conditions. The mechanism by which cells select specific mRNAs for translation is not fully understood, but the exon junction complex (EJC) plays a major role in this process in eukaryotes [4].

The EJC selects the mRNA isoforms to be exported to the cytoplasm and determines whether they are then translated into protein. It also targets transcripts with premature stop codons for degradation via nonsense-mediated decay [5]. The role of the EJC in post-transcriptional regulation of gene expression following myocardial stress has not been investigated but is likely to play a major role in cardiac function. This is because the stresses which lead to heart failure such as hypoxia and mechanical stress also lead to significant changes in gene expression [6]. Understanding the role of the EJC and its associated proteins in response to stress will provide significant new insights into the pathogenesis of heart failure, by identifying potential new targets for the treatment of the disease.

The EJC contains more than 20 different proteins, including the core proteins eukaryotic translation initiation factor 4A isoform 3 (eIF4A3), Y14, mago nashi (Mago), metastatic lymph node 51 (MLN51) and UAP56 which anchor other EJC components to the spliced mRNA [7,8]. eIF4A3 is also the least abundant of the EJC proteins with only 10,000 subunits per cell compared with 40,000 for both Mago and Y14 [9]. This makes eIF4A3 levels the limiting factor in the regulation of gene expression. Therefore, small variations in the expression and distribution of this nucleocytoplasmic shuttling protein will have a profound impact on mRNA processing and potential adaptation to stress. Previous work has shown that in Arabidopsis, there is rapid relocalization of eIF4A3 protein within the domains of the nucleus in response to hypoxic stress [10]. These data indicate that members of the EJC are involved in the stress response in plant cells.

We studied cardiac myocytes to determine whether the subcellular distribution of two members of the EJC (eIF4A3 and Mago) proteins is altered by metabolic stress. We show for the first time that hypoxia or NaN_3_-mediated metabolic stress induced the relocalization of eIF4A3 and Mago preferentially to the cytoplasm. Inhibition of AMP kinase (AMPK) with dorsomorphin (DM) did not inhibit the NaN_3_-mediated relocalization but rather augmented it and accordingly resulted in enhanced cessation of beating. This suggests that the EJC response to metabolic stress is independent of AMPK activation. Knockdown of eIF4A3 with siRNA significantly reduced sarcomere number, cell and nuclear size, and subsequently abrogated myocyte beating. This indicates that eIF4A3 is required for maintaining cardiac contractility and cell architecture. Studying the EJC proteins in cardiomyocytes may therefore provide new insights into the mechanisms which link metabolic stress with cardiac disease following myocardial infarct.

## Materials and methods

### Cell culture and treatments

Myocytes were isolated from the cardiac ventricles of 1–2-day-old rats by sequential collagenase digestion, as previously described [11]. Cells were pre-plated for 1h and 15 min to reduce fibroblasts and other non-myocyte cell contamination and then plated at 2 million cells per 35 mm diameter fibronectin-coated Petri dish. Myocytes cultures were performed in PC1 medium (Lonza) for 24 h minimum, then transferred to Dulbecco-modified Eagle’s medium DMEM-F12:M199 serum-free medium 24 h before treatment with drug or hypoxia.

Sodium azide (NaN_3_, Sigma) was prepared at 1 M in phosphate buffer saline (PBS), and pH of stock solution was adjusted to 7.4. To mimic the energetic stress resulting from hypoxic condition, cells were treated for 4 or 8 h with 50 mM NaN_3_. For AMP-activated protein kinase (AMPK) inhibition, cells were pre-treated for 1 h with 1 µM DM dihydrochloride (Tocris Bioscience) and then incubated further for 4 h in the presence of 50 mM NaN_3_.

Hypoxia was achieved by using an MIC-101 modular incubator chamber (Billups-Rothenberg, Inc, Del Mar, California) which is capable of depleting oxygen down to 0.1% after 10 min flushing at 20 l/min with 5% CO_2_/95% N_2_ pre-mixed gases (BOC), as monitored by a Dräger Pac3500 O_2_ detector. Cultured neonatal rat cardiomyocytes were subjected to acute hypoxic conditions (*p*O_2_ < 0.1%) in the anaerobic chamber at 37°C for various time points ranging from 1 to 18 h, while controls were left in normoxic conditions at 37°C for the same time periods. Oxygen depletion was controlled at the end of each hypoxic cycle with Methylene Blue indicators. Once removed from the 37°C hypoxia chamber, samples were quick chilled on an ice-cold copper plate to limit the likely reversibility of molecular processes induced by hypoxia. Changes induced by hypoxia such as phosphorylation and subcellular relocalization of proteins are transient and can be reversed upon reoxygenation [12].

### Recombinant proteins and antibodies used in the present study

Recombinant GST-eIF4A1 (full sequence) and truncated GST-eIF4A2 (first 100 amino acids) proteins were purchased from Novus Biologicals. eIF4A3 antibodies were raised in rabbit against the first N-terminal either 74 (Sigma Prestige, antibody a) or 20 (antibody b, generous gift from Dr Herve Le Hir, Ecole Normale Superieure, Paris) amino acids. Other antibodies used in the present study are from Abcam (actin, sarcomeric alpha actinin, hif1α, integrin β1), Merck Millipore (Mago), Novus Biologicals (eIF4A1), Sigma (Y14), GenTex (histone H2B) or Cell Signaling (AMPK, pAMPK and polyA-binding protein, PABP1).

### DNA cloning and cell transfection

eIF4A3 protein was cloned using Invitrogen Gateway recombination method. Rat eIF4A3 full-length cDNA clones MGC:125040, pDONR221, Vivid colours pCDNA 6.2/N-term-Em GFP-DEST, Vivid colours pcDNA 6.2/N-term-Em GFP-GW-CAT (GFP expression positive control), and pcDNA™-DEST40 (C-term 6xHis-V5 epitope Tag) were obtained from Thermo-Scientific (Life Technologies Ltd, Paisley, U.K.). Using clone MGC:125040 as a template and attB-containing primers (Forward: 5'-GGGG ACA AGT TTG TAC AAA AAA GCA GGC ATC ATG GCG GCC ACG GCC ACG ATG-3'; Reverse with stop codon : 5'-GGG GAC CAC TTT GTA CAA GAA AGC TGG GTC TCA GAT GAG GTC AGC CAC ATT C-3' or without stop codon 5'-GGG GAC CAC TTT GTA CAA GAA AGC TGG GTC GAT GAG GTC AGC CAC ATT CAT AG-3'), eIF4A3 full-length fragments were amplified by PCR. The resulting attB-containing PCR-generated fragments were used for recombination reaction with pDONR221 (mediated by BP clonase II) to produce Gateway entry clones containing full-length eIF4A3 cDNA in pENTR221. Subsequently, LR clonase II recombination reaction was performed between entry clones and destination vectors pcDNA 6.2/N-Em GFP-DEST and pcDNA-DEST-40, to generate expression clones of eIF4A3 with N-terminal GFP tag (pGFP-eIF4A3-11/12/21) or C-terminal V5 epitope tag (peIF4A3-V5-31), respectively. All cloned DNA sequences were fully checked by sequencing using Source Bioscience (Oxford) sequencing service.

Plasmids were propagated, in DB3.1 Competent Cells with gyrA462 allele, Cat. No. 11782-018, were used to confer resistance to the CCdB toxin and were purified using HiSpeed Plasmid Midi Kit from Qiagen Ltd. (Manchester). Prior to transfection, primary cardiomyocytes were platted 24 h in advance in PC1 media, to reach 50–80% confluency at the time of transfection using JetPei (PolyPlus) reagent. Following the manufacturer’s transfection protocol, 200 μl of 3 μg DNA plasmid:JetPei reagent (1:1) were pre-mixed and added dropwise to each culture well already containing 2 ml of PC1 media. Cells were incubated overnight at 37°C, then media were changed to Dulbecco-modified Eagle’s medium DMEM-F12:M199 (1:4). Transfected cells were identified by GFP-fluorescence (indicating expression of recombinant protein), detected on inverted fluorescent microscope and overexpressed tagged-protein was confirmed by Western blot analysis.

### siRNA-mediated knockdown

eIF4A3 was knocked down in myocytes by passive uptake of Accell (Thermo Scientific Dharmacon) SMART pool rat eIF4A3 siRNA with following sequences: CAAUCAAGAUGUUGGUUUU, CCAUCAAUUUUGUGAAGAA, GGACGAGUCUUUGAUAUGA, CAUUAAACAUGGAAAUUUU. As negative control, SMART pool non-targeting control (NTC) siRNA was used with the following scrambled sequences: UGGUUUACAUGUCGACUAA, UGGUUUACAUGUUUUCUGA, UGGUUUACAUGUUUUCCUA and UGGUUUACAUGUUGUGUGA. Twenty-four hours after plated in PC1 media, myocytes were incubated overnight in 1 ml of Accell delivery media supplemented with 1 µM SMART pool eIF4A3-targeting or NTC siRNA or siRNA buffer only (20 mM KCl, 6 mM HEPES (pH 7.5), and 0.2 mM MgCl_2_, Thermo Scientific Dharmacon). After 16-h incubation, myocyte cultures were toped up with 1 ml of complete PC1 medium and incubated at 0.5 µM siRNA final concentration for further 48, 72, 96 and 120 h before processed to treatment.

### Cell fractionation

For subcellular fractionation of myocytes, the ProteoExtract Subcellular Proteome Kit from Calbiochem (Merck Millipore) was used as described previously [11]. Cellular proteins were sequentially extracted into four compartments: cytosolic, membrane/organelles, nuclei and cytoskeleton. The accuracy of the fractionation method was verified with antibodies to well-documented subcellular markers.

### Western blotting

Myocytes were rinsed with PBS and scraped from the Petri dishes in 1X RIPA lysis buffer (RIPA buffer 10X, Cell Signaling) supplemented with 0.1% SDS, protease inhibitor cocktail III (Calbiochem) and phosphatase inhibitor cocktail (Sigma). DC Protein Assay from BioRad was used to determine total protein. Samples were treated with β-mercaptoethanol and heated to 95°C for 5 min. Proteins were separated by SDS/PAGE and transferred to PVDF membrane (BioRad). Blots containing either whole cell lysates or fractionated cells were probed with primary antibodies: eIF4A3 1:500 (antibody a) or 1:5000 (antibody b); all other antibodies at 1:1000 except actin (1:5000). Horseradish peroxidase-conjugated secondary antibodies anti-mouse, anti-rabbit or anti-goat (Thermo-Scientific, Life Technology) were used to visualize proteins by enhanced chemiluminescence (ECL) solutions of various sensitivity (Thermo Scientific West Pico & Femto; BioRad Clarity), depending on the strength of the chemiluminescent signal. The bands corresponding to the various proteins were detected on ImageQuant LAS 4000 (GE Healthcare) and quantified by densitometry using ImageJ software. Protein bands were standardized to total protein as previously described [13].

### Immunocytochemistry

For immunocytochemical staining, cells were washed twice in PBS, fixed in 4% paraformaldehyde for 5 min and then covered with 70% ethanol for storage at −20°C. When immunostained, cells were rehydrated in PBS and then stained with antibodies as described previously [11]. Primary antibodies were used at a dilution of 1:500, except for Mago (1:250) and eIF4A3 (antibodies a and b at 1:250 and 1:2000 respectively). Alexa fluorophore-conjugated secondary antibodies (Thermo-Scientific, Life Technology) were used at a dilution of 1:500. Fluorescently labelled cells were viewed using Zeiss Axioscope fluorescence microscope (Zeiss, Cambridge, U.K.), and images were captured using an Axiocam digital camera system (Zeiss) and Axiovision image analysis software (version 4.7, Zeiss). Images from immunocytochemistry were analysed on ImageJ and ratio of cytosolic over nuclear fluorescence was obtained by subtracting the background fluorescence measured in several points next to each cell. The corrected total cell fluorescence (CTCF) intensity is measured according to the formula: CTCF = integrated intensity – (area of selected cell × mean fluorescence of background readings).

### Time lapse live cell imaging microscopy

Live cells in bright field and fluorescently labelled cells were observed under a Nikon eclipse (TE2000-U) time-lapse inverted microscope (Nikon Instruments).

### Sarcomere damage analysis

The number of intact sarcomeres was determined by staining the cells for α-actinin and counting the number of sarcomeres in a 10 × 10 μm box in four areas chosen at random per image as described previously [14]. At least four images were analysed per condition, and data are shown where *n* = number of images.

### Statistical analysis

For the experiments described here, at least three separate primary cultures were averaged. Each culture used approximately 30 neonatal hearts. All values are means ± SEM. All values of significance were calculated using the appropriate comparisons: one-way analysis of variance or the Student’s unpaired *t*-test. Differences among means were considered significant at *P*<0.05. Data were analysed using Microsoft Excel and Minitab statistical software.

## Results

### Subcellular fractionation of cultured neonatal myocytes

**Figure 1:**
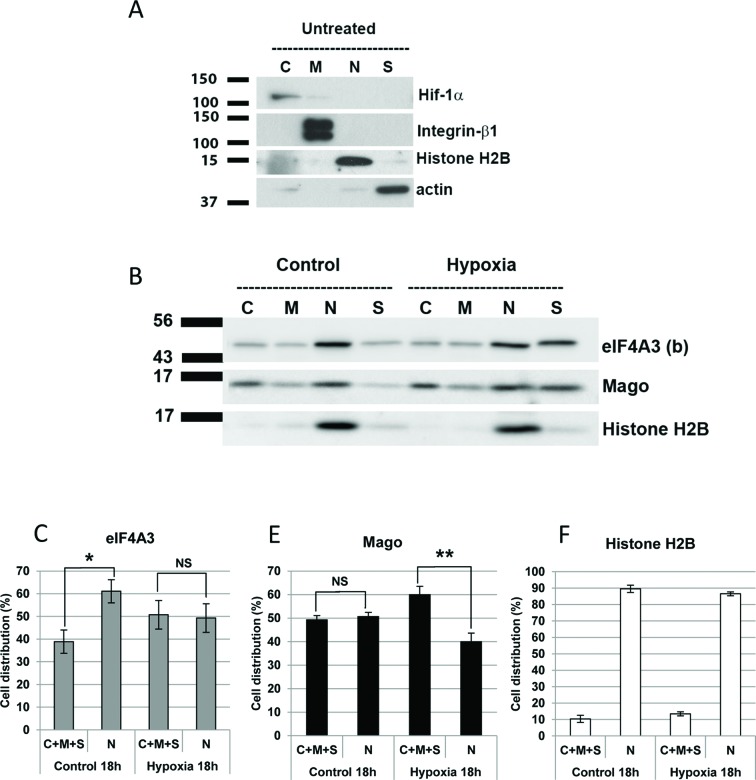
Hypoxia induces the subcellular relocalization of core EJC proteins eIF4A3 and Mago (**A**) Western blot of Hif-1α, integrin-β1, histone H2B and actin following subcellular fractionation of cultured rat neonatal cardiac myocytes. These have been used as the marker of the cytoplasmic (C), membrane (M), nuclear (N) and cytoskeletal (S) pool of protein respectively, to verify the fractionation process. (**B**) Western blot to assess the subcellular distribution of eIF4A3 and Mago following treatment of cultured neonatal rat cardiac myocytes with 18 h of hypoxia. Histone H2B has been used as a marker of the nuclear fraction. (**C–F**) Quantification of subcellular distribution of eIF4A3, Mago and histone H2B in the nucleus and cytoplasm (C + M + S) following treatment of myocytes with 18 h of hypoxia; *P<0.05 and **P<0.01, *n*=4. NS: not significantly different.

In the present study, we set out to study the subcellular movement and potential function of the EJC proteins in response to hypoxia and metabolic stress since these are major causes of cardiovascular disease in man. To study how metabolic stress affects EJC function, we have examined the effect of hypoxia and the respiratory inhibitor sodium azide (NaN_3_) in cultured neonatal myocytes. Using a detergent-based subcellular fractionation method [15], we fractionated myocytes into cytosolic, membrane, nuclear and cytoskeletal components. Figure 1(A) shows that Hif-1α localized to the cytosol, β_1_-integrin to the membrane, histone H2B in the nucleus and actin in the cytoskeleton. Once the methodology was verified using proteins with a known subcellular location, we followed by analysis of changes in the pattern of protein localization for EJC components as well as other marker proteins.

### Specificity of eIF4A3 antibodies

eIF4A3 shares 65% sequence identity with cytoplasmic homologues eIF4A2 and eIF4A1, so we wanted to validate our antibodies before use. We detected purified human recombinant GST (gluthatione S-transferase)-tagged eIF4A isoforms (Novus Biologicals) in Western blots (Supplementary Figure S1). We found that the custom-made antibody raised against the first 20 amino acids of eIF4A3 (antibody b) was the most specific in detecting isoform 3 of eIF4A proteins, whereas the Prestige antibody (first 74 amino acids, antibody a) cross-reacted with cytoplasmic isoform 2 (Supplementary Figure S1, panel A), albeit more weakly than the eIF4A1 antibody. This demonstrates that the specificity of the antibody for isoform 3 of eIF4A proteins is governed by the size of the N-terminal peptide sequence used as immunogen.

### Hypoxia induced relocalization of EJC proteins in cardiac myocytes

Previous work by one of the authors here has shown that eIF4A3 protein fused to green fluorescent protein (GFP) is highly dynamic in Arabidopsis cells, and rapidly changes its pattern of localization in the nucleus under hypoxic conditions [10]. In the present study, we set out to determine whether endogenous EJC proteins would alter their subcellular distribution in response to hypoxia in cardiac myocytes. Western blot analysis on cell fractions was performed with anti-eIF4A3(b) and anti-Mago antibodies. Core EJC proteins eIF4A3 and Mago relocalized outside the nucleus to the cytoplasm and/or the cytoskeleton following 18 h of hypoxia (<0.1% O_2_) as seen in Figure 1(B)–(D). This movement was not observed at earlier time points, including 12 h of hypoxia (data not shown). Histone H2B, a nuclear protein associated with chromatin, was used as an internal control and showed no change in localization in response to hypoxia (Figure 1B and E).

### Sodium azide treatment activates AMP kinase similar to hypoxia in cardiac myocytes

**Figure 2:**
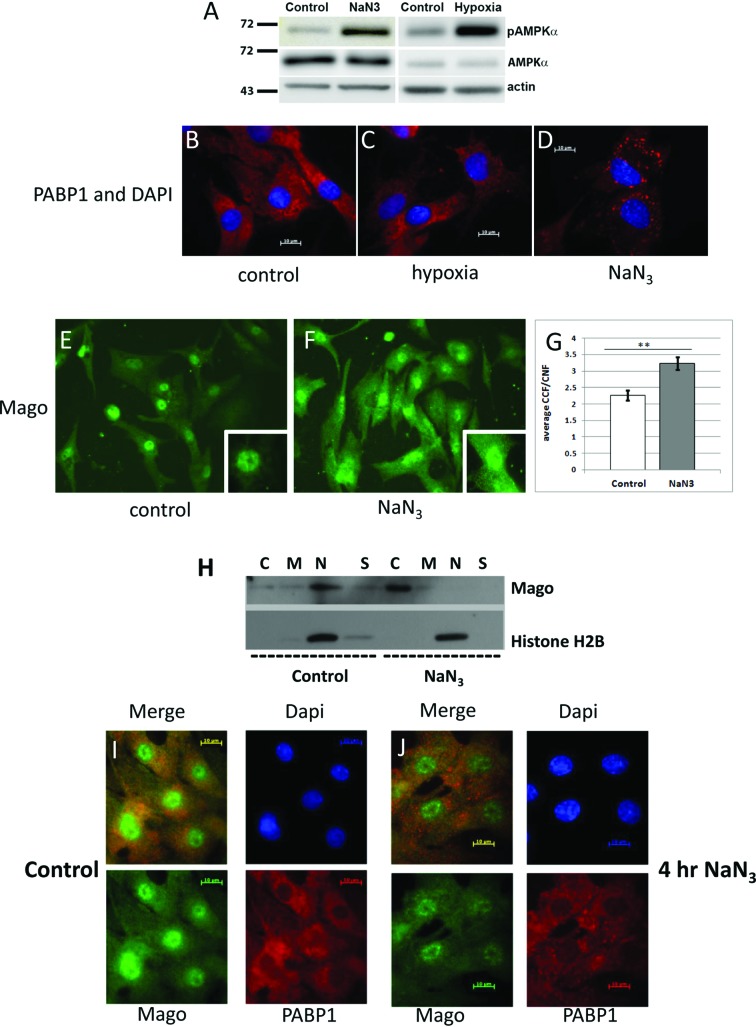
Metabolic stress induced by sodium azide is similar to hypoxia in activating AMPK and promoting the cytoplasmic relocalization of Mago (**A**) Western blot of total and phosphorylated AMPK in cardiac myocytes treated for 8 h with 50 mM sodium azide (NaN_3_) or 12 h with hypoxia. Actin was used as a loading control. (**B–D**) Immunostaining of cardiac myocytes with the stress granule (SG) marker PABP1 (red) and DAPI (blue) following hypoxia and NaN_3_ treatments. Panel (D) shows the formation of SGs only with NaN_3_-treated cells. (**E**) Immunostaining of cardiac myocytes for Mago (green) in control (**E**) and NaN_3_-treated (**F**) cells. (**G**) Quantification of the immunofluorescence of Mago in control and NaN_3_-treated cells where *P*<0.01, *n*=25 cells. (**H**) Western blot to assess the subcellular distribution of Mago and histone H2B following NaN_3_ treatment. (**I–J**) Immunostaining of cardiac myocytes for Mago (green), PABP1 (red) and DAPI (blue) in control (I) and in cells treated with NaN_3_ (J), showing SG formation. **represents the difference between control and NaN_3_-treated cells.

Studying the effect of metabolic stress by hypoxia presents a number of technical challenges, including rapid reoxygenation during multiple sample processing. As a result, we determined whether the respiratory inhibitor NaN_3_ could mimic the effect of hypoxia as a mean of studying metabolic stress in cardiac myocytes. The myocytes were treated with NaN_3_ to induce chemical hypoxia and this was compared directly with hypoxia. To analyse the hypoxia and chemically induced anoxia treatments at the molecular level, we measured activation of the metabolic sensor AMPK by determining its phosphorylation at Thr^172^ in response to treatments. Both hypoxia and NaN_3_ increased AMPK phosphorylation as shown in Figure 2(A). The treatments did not change total AMPK or actin protein levels.

### Sodium azide induces stress granules in cardiac myocytes

Metabolic stress sometimes results in the formation of SGs [16], so myocytes were immunostained for SGs marker PABP1 following either hypoxia or NaN_3_ treatment (Figure 2B–D). Distinctive cytosolic SGs were formed after 4-h treatment of myocytes with 50 mM NaN_3_, as shown in Figure 2(D). No such SGs were detected after 12 h of hypoxia (Figure 2C).

### Mago relocalized to the cytoplasm after metabolic stress

Based on the above data, we used NaN_3_ as a substitute for hypoxia to study the EJC in cardiac myocytes. Cells were treated with 50 mM NaN_3_ for 4 h and immunostained for Mago (Figure 2E and F). There was an apparent redistribution of Mago to the cytoplasm following treatment. Following quantification of the background-corrected fluorescence, there was a significant 43% increase in the cytoplasmic to nuclear ratio in cardiac myocytes following NaN_3_ treatment (**represents control myocytes versus NaN_3_ treatment, *P*<0.01, *n*=25), Figure 2(G). Western blot analysis showed that Mago relocated from the nuclear fraction to the cytoplasm following NaN_3_ treatment (Figure 2H). Histone H2B was used as a fractionation control and showed no movement in response to treatment. Finally, to determine whether the relocalizing Mago protein was associated with SGs, cells were co-stained with the SG marker PABP1 (Figure 2I–J). Mago did not co-localize with PABP1 in SGs following NaN_3_ treatment.

### Metabolic sensing of EJC proteins is not AMP kinase dependent

**Figure 3:**
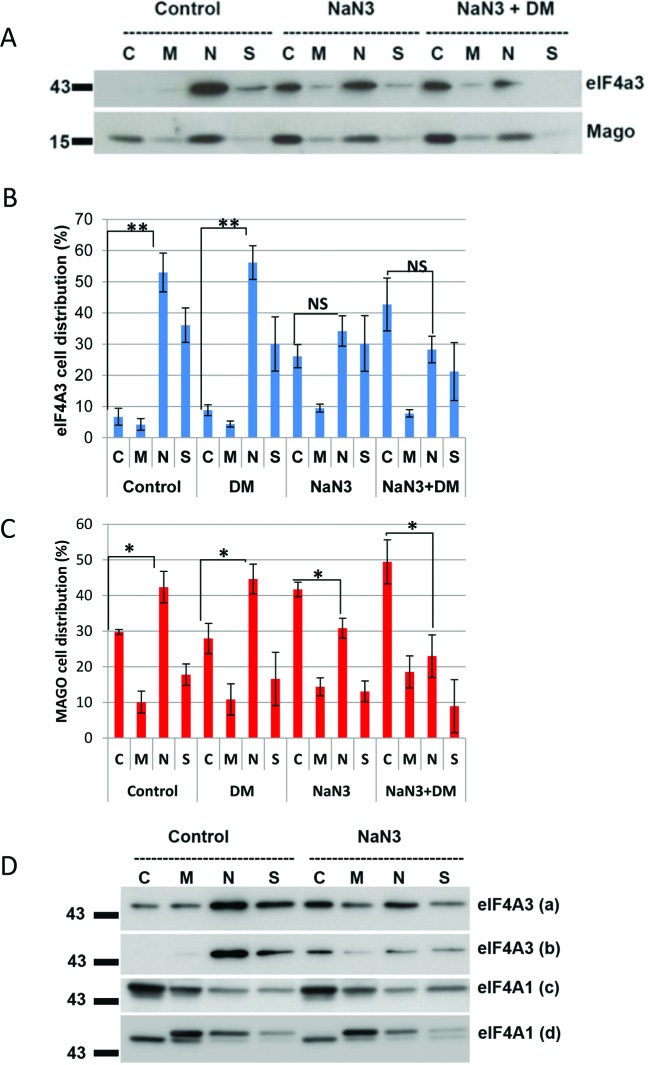
Synergistic effect of sodium azide and AMPK inhibitor DM on the EJC protein eIF4A3 and Mago subcellular relocalization (**A**) Western blot of eIF4A3 and Mago following subcellular fractionation of cardiac myocytes in control cells, cells treated with 50 mM NaN_3_ and in cell treated with NaN_3_ plus 1 µM DM. Quantification of subcellular distribution of eIF4A3 (**B**) and Mago (**C**) in control cells and cells treated with DM, NaN_3_ and DM + NaN_3_. **P<0.01 and *P<0.05 compared with controls; NS=non-significant. *N*=4 separate cultures. (**D**) Western blot to test the specificity of eIF4A3 and eIF4A1 antibodies following NaN_3_ treatment. Cytosolic (C), membrane (M) nucleus (N) and cytoskeleton (S) (labels (a) and (b) represent two eIF4A3 antibodies; (c) and (d) show two eIF4A1 antibodies).

Since AMPK is activated by metabolic stress in cardiac myocytes (Figure 2A), we examined whether the subcellular movement of EJC components in response to NaN_3_ might be dependent on the enzyme. To test this, myocytes were treated with 50 mM NaN_3_ in combination with 1 µM DM to inhibit AMPK activation. In untreated cells, a majority of eIF4A3 and Mago protein is localized to the nucleus (Figure 3A). However, following NaN_3_ treatment both proteins are significantly reduced in the nucleus with a translocation to the cytosolic fraction (Figure 3B and C). NaN_3_ in combination with DM further augmented the cytosolic translocation of eIF4A3 and Mago, whilst AMPK inhibitor DM alone had no effect.

Concomitant with these biochemical observations, the dual treatment of NaN_3_ and DM completely arrested myocyte contractility while NaN_3_ treatment alone did not completely inhibit contraction (please see the video files in the supplementary material). These data suggest that inhibition of AMPK enhances the nuclear to cytoplasmic translocation of both eIF4A3 and Mago proteins in response to metabolic stress.

### eIF4A1 does not respond to metabolic stress

To determine whether the cytoplasmic translocation observed in response to metabolic stress was unique to the eIF4A protein isoform 3, samples were also probed for eIF4A1 (the cytoplasmic isoform involved in translation initiation) following NaN_3_ treatment. As expected, a majority of eIF4A1 protein was in the cytoplasm and showed no mobility in response to metabolic stress while eIF4A3 showed a clear movement from nucleus to cytoplasm (Figure 3D).

### Tagged eIF4A3 has aberrant distribution and does not respond to metabolic stress

To further study the function and activity of eIF4A3 protein, we transfected cardiac myocytes with plasmids containing the full-length rat sequence of eIF4A3 tagged to GFP on the N-terminus or V5 on the C-terminus. As a control, cells were non-transfected (Supplementary Figure S1B, lane 1) or transfected with GFP-Cat (chloramphenicol acetyl transferase). The cells were then fractionated into cytosol (C), membrane (M), nucleus (N) and cytoskeleton (S). By Western blot, we confirmed that both recombinant GFP-tagged eIF4A3 (Supplementary Figure S1B, lane 3) and GFP-Cat (Supplementary Figure S1B, lane 2) were expressed in myocytes following transfection.

GFP N-terminal tagged eIF4A3 had an aberrant subcellular distribution in cardiac myocytes with a majority of the protein in the cytosolic fraction compared with mostly nuclear for the native protein (Supplementary Figure S2, panel A). Following metabolic stress induced by NaN_3_, there was no change in the subcellular distribution of the N-terminally tagged eIFaA3 protein, whilst the native protein showed a clear nuclear to cytoplasmic shift following treatment (Supplementary Figure S2, panel A). The gel has been probed with eIF4A3 antibody b. Western blot of cardiac myocytes transfected with C-terminal tagged V5 (Supplementary Figure S2, panel B). Following NaN_3_ treatment, there was no change in the subcellular distribution of the V5 C-terminal tagged-eIF4A3 (gel probed with V5 antibody), whilst the native protein showed a clear nuclear to cytoplasmic shift (Supplementary Figure S2, panel B)_._

### eIF4A3 genetic knockdown impaired myocyte contractility and sarcomeric structure

**Figure 4:**
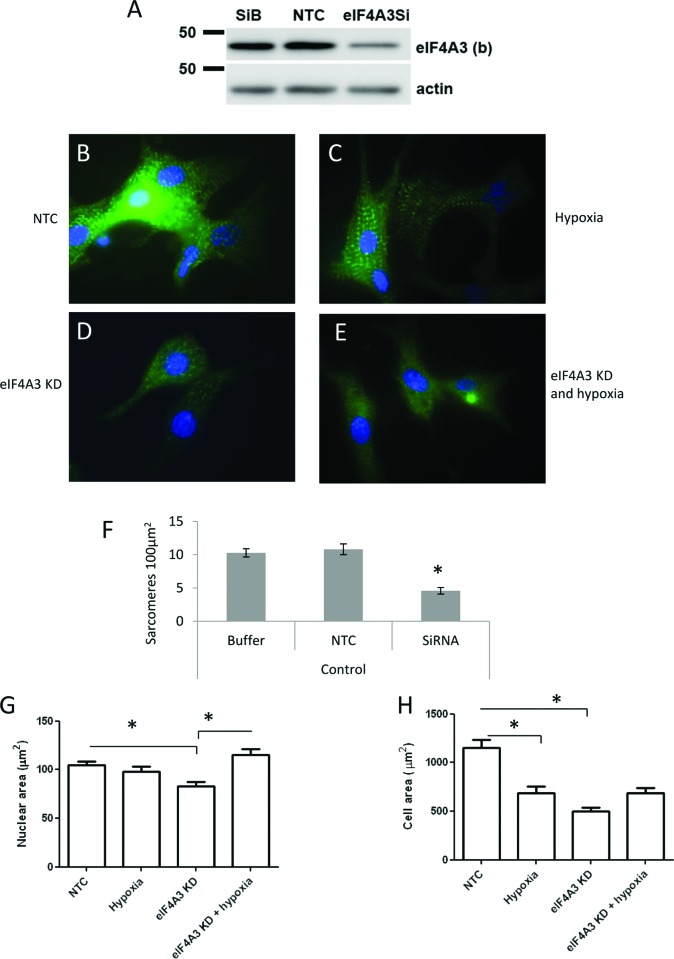
siRNA knockdown of eIF4A3 results in reduced myocyte sarcomeres, cell and nuclear size (**A**) Western blot showing the level of eIF4A3 protein in cardiac myocytes where the cells have been transfected with just the siRNA buffer (SiB), with NTC siRNA or transfected with eIF4A3 siRNA to knockdown the level of eIF4A3. Immunofluorescence of α-actinin (green) and DAPI (blue) in NTC siRNA treated-cells (**B**), 18 h of hypoxia (**C**), cells where eIF4A3 has been knocked down with siRNA (**D**) and those with siRNA and hypoxia (**E**). (**F**) Quantified cardiac myocyte sarcomere numbers in cells from control and siRNA treated; *N*=3 separate experiments; *P<0.05 compared with NTC siRNA. (**G**) Measurement of cell area in the four treatment groups, with and without siRNA or hypoxia. NTC versus hypoxia, *P<0.05, *n*=40–50 cells; NTC versus eIF4A3kd *P*<0.05, *n*=40–50 cells. (**H**) Measurement of nuclear area in the four treatment groups, with and without siRNA or hypoxia. NTC versus eIF4A3kd, *P*<0.05, *n*=40–50 cells; eIF4A3kd versus eIF4A3kd with hypoxia, *P<0.05, *n*=40–50 cells. KD= knockdown

To determine the function of the EJC core component eIF4A3 in cardiac myocytes, the protein was knocked down by siRNA treatment (Figure 4A). 96 h post-siRNA treatment resulted in cessation of myocyte contractility. After this period, there was a significant reduction in eIF4A3 expression by up to 70% and an alteration of cell morphology in which the cells became more elongated, with fewer intact sarcomeres (Figure 4D). Myocyte sarcomere number was quantified and showed a significant reduction in sarcomere density following eIF4A3 knockdown as shown in Figure 4(F). To further assess cell morphology, both cell and nuclear area was measured with and without hypoxia or eIF4A3 knockdown. Myocyte cell area was significantly reduced by both hypoxia and eIF4A3 knockdown. Control versus hypoxia *P<0.05 and control versus eIF4A3kd *P*<0.05, *n*=40–50 cells. eIF4A3 knockdown significantly reduced nuclear area NTC versus eIF4A3kd, *P*<0.05, *n*=40–50 cells. However, this was prevented in the presence of hypoxia. eIF4A3kd versus eIF4A3kd with hypoxia *P<0.05, *n*=40–50 cells.

## Discussion

The EJC has been studied in simple model systems including transformed cell lines; however, very little is known about the expression, activity and function of this important complex in more specialized cell types such as cardiac myocytes. In the present study, we show for the first time that members of the EJC relocalize to the cytoplasm following metabolic stress induced by both hypoxia and sodium azide treatment in cardiac myocytes. This metabolic stress was designed to mimic some of the changes which occur in the heart following a myocardial infarct and therefore these data may be relevant to disease. The relocalization is independent of AMPK activity and inhibition of the metabolic sensor actually augmented the effect of metabolic stress on the EJC. These observations are somewhat distinct from those we previously described in Arabidopsis in which hypoxia caused a rapid relocalization of eIF4A3 to nuclear speckles [10]. The EJC response to hypoxia in myocytes is considerably slower and may be a specific feature of cardiomyocytes which are usually resistant to metabolic stress. The EJC components usually cycle back into the nucleus after entering the cytoplasm and the accumulation of eIF4A3 and Mago here is likely to alter both the selection and translation of mRNA transcripts into protein. The EJC has been shown to interact with the mTOR signalling pathway involved in nutrient, energy and stress-sensing in mammalian cells, providing a potential mechanistic link between the cellular stress-response and altered translation efficiency of spliced over non-spliced mRNAs [17].

In the heart, atherosclerosis is the main cause of hypoxia and has severe clinical implications in being the major cause of heart failure in man [18]. Hypoxia and the subsequent metabolic stress result in the activation of the metabolic sensor AMPK which is cardioprotective [19]. Interestingly, the response of the EJC to metabolic stress, namely its accumulation outside the nucleus into the cytosol, was completely independent of AMPK and inhibitors of this enzyme actually augmented the response to metabolic stress. These data strongly suggest that the EJC reacts to the magnitude of metabolic stress and is independent of AMPK in cardiac myocytes. AMPK is activated by increases in the AMP:ATP which can be caused by stresses which inhibit ATP synthesis or increase ATP usage [20]. Our data show that chemical hypoxia induced by NaN_3_ also activates AMPK and this has been previously shown to increase cellular glucose uptake through the up-regulation of GLUT-4 receptors in myocytes [21]. This may indicate that both hypoxia and NaN_3_ result in similar degrees of metabolic stress. eIF4A3 is an ATP-dependent helicase and therefore it is possible that the alteration to its localization during metabolic stress is a direct response to depleted cellular ATP levels [22].

During periods of stress, specific mRNAs may accumulate in cytoplasmic structures called SGs [23]. Proteins involved in RNA processing, cell adhesion, signalling and growth also integrate within these structures. SGs may be protective in the short term because they allow proteins involved in cell survival to be expressed and regulated during stress [23]. However, SGs have also been implicated in the pathogenesis of diseases including cancer, neurodegeneration and autoimmune diseases suggesting that in the long term, they may be detrimental [23–25]. Interestingly, in cardiac myocytes, we found that sodium azide but not hypoxia induced SGs despite both of them activate AMPK. This suggests that SG formation pathways may not necessarily be linked to metabolic stress alone. The EJC cytoplasmic relocation occurs whether or not SGs are present. A number of RNA processing proteins associate with cytoplasmic SGs and influence the stress response [23] including MLN51 [26]. MLN51 is also involved in the formation of a new type of cytoplasmic RNA granule which is distinct from stress-granules or p-bodies [27]. Our data show that eIF4A3 and Mago both accumulate in the cytoplasm in response to metabolic stress but we were unable to observe the association of Mago with the SGs marker PABP1. Whether they associate with p-bodies or not, their physiological function needs to be investigated in specialized cells like those of the heart. What seems clear is that the EJC members appear to have an ability to sense hypoxia/respiratory stress upstream of metabolic sensing pathways.

Our data show that the movement of eIF4A3 and Mago occur in synchrony suggesting that components of the EJC can act as a single unit; however, it cannot be ruled out that they are capable of acting separately. We selected eIF4A3 for knockdown in cardiac myocytes because this RNA helicase is known to be essential for mRNA quality control in human cells [28,29]. eIF4A3 is also the least abundant of the EJC proteins with only 10,000 subunits per cell compared with 40,000 for both Mago and Y14 [9]. This makes eIF4A3 levels the limiting factor in the regulation of gene expression. Therefore, small variations in the expression of this protein would have had a profound impact on mRNA processing and potential adaptation to stress. We showed that knockdown of eIF4A3 in myocytes by 70% produced a major phenotype. After 96 h, the myocytes stopped beating and the number of intact sarcomeres significantly decreased along with significant changes in cell shape. We have previously shown that the loss of sarcomeres is associated with decreased myocyte function [30]. These data strongly suggest that eIF4A3 plays an important role in the heart, including contractility and sarcomerogenesis. Mutation in another EJC component CDC174 results in loss of skeletal muscle myofibrils, suggest a strong link between EJC activity and striated muscle structure [31].

Our data show that knockdown of eIF4A3 in myocytes resulted in reduced cell and nuclear size. The reduction in nuclear size usually a hallmark of cellular apoptosis [32]. However, this is very unlikely to be the case here since the combination with hypoxia actually reversed the reduction induced by eIF4A3 knockdown. This probably suggests that eIF4A3 is involved in the regulation of cell architecture as illustrated by the concomitant reduction in cell size. The reduction in cell size by eIF4A3 knockdown mimicked the effect induced by hypoxia alone. The reduction in cell size following hypoxia in myocytes is usually the result of rigour induced by reduced cellular ATP [33]. It seems that loss of eIF4A3 has a dramatic effect on myocyte cell architecture and function.

Interestingly, recombinant eIF4A3 tagged on either the C- or N-terminus had aberrant localization in cardiomyocytes and did not respond to energetic stress by cytoplasmic relocation. This may suggest that tagging of the protein altered its metabolic sensing function and possibly its interaction with other proteins and members of the EJC in cardiomyocytes. We have previously shown that recombinant proteins tagged on either the C- or N-termini can result in abnormal protein function and localization [34].

In the present study, we investigated the role of the EJC in post-transcriptional regulation of gene expression following myocardial stress caused by hypoxia and NaN_3_. Our results provide the first evidence that EJC components are likely to play a major role in cardiac function. This is because the stresses which lead to heart failure such as hypoxia and mechanical stress also lead to significant changes in gene expression [6]. Cellular processes such as apoptosis are significantly increased in heart failure [35] and the EJC members regulate this via alternative splicing of apoptotic regulators [36]. Further work needs to be done to determine the role of the EJC components in cardiac adaptation and disease. It is likely that such studies will require inducible models since the early knockdown of the EJC components will most likely be lethal. However, studies such as those presented here provide a first step in understanding the potentially complex role of the EJC in health and disease.

The EJC provides a potential therapeutic target for regulating alternative splicing to change the initiation and progression of disease. Small molecules can be used to affect the activities of RNA-binding proteins via phosphorylation, and can have substantial effects on splicing, processing and translation efficiency of targeted transcripts [37]. In conclusion, we show for the first time that metabolic stress significantly disrupts the nucleocytoplasmic shuttling of eIF4A3 and Mago which is enhanced by AMPK inhibition. siRNA knockdown of eIF4A3 disrupts myocyte sarcomere structure and contractile activity. This suggests that the members of the EJC play a prominent role in myocyte and heart function.

## Abbreviations

AMPK: AMP kinase
CTCF: corrected total cell fluorescence
DM: dorsomorphin
eIF4A3: eukaryotic translation initiation factor 4A isoform 3
EJC: exon junction complex
GLUT-4: glucose transporter 4
mTOR: mammalian target of rapamycin
MLN51: metastatic lymph node 51
NTC: non-targeting control
PABP1: polyA-binding protein
SG: stress granule
